# Exaggerated effects of particulate matter air pollution in genetic type II diabetes mellitus

**DOI:** 10.1186/1743-8977-11-27

**Published:** 2014-05-30

**Authors:** Cuiqing Liu, Yuntao Bai, Xiaohua Xu, Lixian Sun, Aixia Wang, Tse-Yao Wang, Santosh K Maurya, Muthu Periasamy, Masako Morishita, Jack Harkema, Zhekang Ying, Qinghua Sun, Sanjay Rajagopalan

**Affiliations:** 1 Department of Physiology, Hangzhou Normal University, Hangzhou, China; 2 Wexner Medical Center, The Ohio State University, Columbus, OH, USA; 3 Division of Cardiovascular Medicine, The Affiliated Hospital of Chengde Medical College, Chengde, China; 4 Department of Environmental Health Sciences, University of Michigan, Ann Arbor, MI, USA; 5 Center for Integrative Toxicology, Michigan State University, Lansing, MI, USA; 6 Division of Cardiovascular Medicine, University of Maryland, Baltimore, MD, USA

**Keywords:** Diabetes, Particulate matter, Thermogenesis, Inflammation

## Abstract

**Background:**

Prior experimental and epidemiologic data support a link between exposure to fine ambient particulate matter (<2.5 μm in aerodynamic diameter, PM_2.5_) and development of insulin resistance/Type II diabetes mellitus. This study was designed to investigate whether inhalational exposure of concentrated PM_2.5_ in a genetically susceptible animal model would result in abnormalities in energy metabolism and exacerbation of peripheral glycemic control.

**Methods:**

KKay mice, which are susceptible to Type II DM, were assigned to either concentrated ambient PM_2.5_ or filtered air (FA) for 5–8 weeks via a whole body exposure system. Glucose tolerance, insulin sensitivity, oxygen consumption and heat production were evaluated. At euthanasia, blood, spleen and visceral adipose tissue were collected to measure inflammatory cells using flow cytometry. Standard immnunohistochemical methods, western blotting and quantitative PCR were used to assess targets of interest.

**Results:**

PM_2.5_ exposure influenced energy metabolism including O_2_ consumption, CO_2_ production, respiratory exchange ratio and thermogenesis. These changes were accompanied by worsened insulin resistance, visceral adiposity and inflammation in spleen and visceral adipose depots. Plasma adiponectin were decreased in response to PM_2.5_ exposure while leptin levels increased. PM_2.5_ exposure resulted in a significant increase in expression of inflammatory genes and decreased UCP1 expression in brown adipose tissue and activated p38 and ERK pathways in the liver of the KKay mice.

**Conclusions:**

Concentrated ambient PM_2.5_ exposure impairs energy metabolism, concomitant with abnormalities in glucose homeostasis, increased inflammation in insulin responsive organs, brown adipose inflammation and results in imbalance in circulating leptin/adiponectin levels in a genetically susceptible diabetic model. These results provide additional insights into the mechanisms surrounding air pollution mediated susceptibility to Type II DM.

## Background

Chronic cardiometabolic disorders such as type 2 diabetes mellitus (Type II DM) have become an uncontrolled epidemic and a burgeoning cause of morbidity and mortality worldwide [1]. As a consequence of rapid increase in combustion of fossil fuels for power generation and transportation, air pollution (indoor and outdoor) has been deemed a major risk factor for death and disability particularly in rapidly developing countries of the world. The cardiovascular and likely metabolic risks posed by inhaled pollutants are primarily mediated by particulate matter content. Both epidemiologic studies and experimental evidence have provided compelling associations between particulate matter, especially particles <2.5 μm in aerodynamic diameter (PM_2.5_), and cardiometabolic disease [2].

Type II DM and its complications are widely believed to result from unbridled inflammatory pathways in insulin-responsive tissues such as the liver, adipose tissue, skeletal muscle and vasculature [3-5]. Adipose tissue functions as an important endocrine and metabolic organ and controls energy balance and lipid homeostasis. White adipose tissues, including visceral adipose tissue (predominantly epididymal and mesenteric in rodents) and subcutaneous adipose tissue (inguinal), serve as the principal energy reservoirs, while brown adipose tissue (BAT) is specialized for energy expenditure and thermogenesis by metabolizing fatty acids and generating heat. PM_2.5_ exposure has been previously demonstrated to result in an increase in macrophages in visceral fat depots with a shift to a pro-inflammatory phenotype characterized by an increase in F4/80 macrophages and a pro-inflammatory “M1 phenotype” typified by an increase in TNFα, IL-6 and a decrease in IL-10, MgI1 gene expression in a diet-induced obesity model [6]. We have also noted multiple abnormalities in mitochondrial rich BAT with PM_2.5_ exposure over long durations in C57BL/6 mice, accompanied by increase in excess oxidative and nitrosative stress [7]. However, it still remains unknown if PM_2.5_ accelerates diabetes development in a genetically susceptible model and if PM_2.5_ influences parameters of energy metabolism. In this study, we examined the hypothesis that inhalation of concentrated PM_2.5_ would result in abnormalities in energy metabolism and exacerbate peripheral glycemic control through inflammatory mechanisms in a genetically susceptible mouse model of Type II DM.

## Methods

### Animals and animal care

KKay mice (5-week-old) were purchased from Jackson Laboratories (Bar Harbor, ME). All mice were maintained at 21°C on a 12-h light/12-h dark cycle with free access to water and food. The protocols and the use of animals were approved by and in accordance with the Ohio State University Animal Care and Use Committee.

### Ambient whole-body inhalational protocol and groups

KKay mice were exposed by inhalation to either filtered air (FA) or concentrated ambient PM_2.5_ for 6 h/d, 5 d/wk from December 28 2011 to February 28, 2012, for a total of 5weeks or 8weeks, in an exposure system (“Ohio Air Pollution Exposure System for Interrogation of Systemic Effects” located at the Ohio State University Animal Facility in Columbus). This exposure system is a mobile trailer versatile aerosol concentration enrichment system which allows concentration of ambient particles 10 ~ 12 folds. Animal exposure and monitoring of the exposure environment and ambient aerosol were performed as previously described [6,8]. For ease of identification, the animal groups were named 5WK-FA, 5WK-PM, 8WK-FA and 8WK-PM (n = 7-8/group). Unless otherwise indicated, PM_2.5_ refers to concentrated ambient particles in this study.

### PM_2.5_ concentration and composition in the exposure chamber

To calculate exposure mass concentrations of PM_2.5_ in the exposure chambers, samples were collected on Teflon filters (PTFE, 37 mm, 2 μm pore; PALL Life Sciences, Ann Arbor, MI) and weighed before and after sampling in a temperature- and humidity-controlled weighing room using a Mettler Toledo Excellence Plus XP microbalance. Weight gains were used to calculate exposure concentrations. Elemental composition was determined by high-resolution inductively coupled plasma-mass spectrometry (ICSP-MS) (ELEMENT2, Thermo Finnigan, San Jose, CA).

### Measurements of blood glucose homeostasis and insulin sensitivity

Before and subsequent to the exposure to FA or PM_2.5_, mice were fasted for intra-peritoneal glucose tolerance testing (IPGTT) or insulin tolerance test (ITT) as previously described. Briefly, mice were fasted overnight and dextrose (2 mg/g body weight) was injected intra-peritoneally for IPGTT. Blood sample was collected from the vena caudalis and blood glucose measurement was conducted with an Contour Blood Glucose Meter (Bayer, Mishawaka, IN) at baseline, and 30, 60, 90, and 120minutes after the dextrose injection. When ITT was performed, insulin (0.5 U/kg) was administered by intra-peritoneal injection after 4.5hours fasting. Blood glucose measurement was conducted in the same way as IPGTT with the same Contour Blood Glucose Meter at baseline, and 30, 60, 90, and 120minutes after insulin injection.

Adiponectin and leptin levels were determined by the mouse adiponectin kit (PerkinElmer, Boston, MA) or leptin quantification kit (Abcam, Cambridge, MA) following the manufacturer’s instructions.

### Oxygen consumption and heat production measurement

The mice were isolated in a semi-sealed cage, and the inner air was aspirated at a constant volume/min. Oxygen consumption, CO_2_ production, respiratory exchange ratio and heat production were measured simultaneously using a computer-controlled, open-circuit Oxymax/CLAMS System (Columbus Instruments, Columbus, OH). Each mouse was measured individually in a resting state at 22°C in the presence of food and water. Measurements were taken for a 24-h period, including a 12-h light cycle and a 12-h dark cycle. Data were normalized to body weight.

### Immunoblotting

Protein levels were determined by western blot. Tissues of brown adipose and liver were homogenized with M-PER Mammalian protein extraction reagent (Thermo Scientific, Rockford, IL) on ice. Equal quantities of proteins from these tissues were loaded and separated by 10% SDS-PAGE. Following transfer to immobilon-P polyvinylidene difluoride (PVDF) membrane and blocking with 5% nonfat milk, the blots were incubated with different primary antibodies: UCP1 (Abcam, Cambridge, MA), P-AKT (phosphorylation at Ser473)/AKT, PI3K, P-AMPK (phosphorylation at Thr172)/AMPK, MAPK pathway proteins (Cell Signaling Technology, Danvers, MA). The immunoblots were then incubated with a secondary antibody conjugated with horseradish peroxidase and visualized with enhanced chemiluminescence, and the autoradiograph was quantitied by densitometric analysis with ImageJ software. β-actin was used as control reference.

### Flow cytometric evaluation of inflammation in blood/tissues

Epididymal white adipose tissue (epiWAT) was excised, minced, and digested with collagenase type II, and the stromal vascular fraction isolated as described previously. Spleens were isolated, homogenized and suspended in PBS. Bone marrow derived cells were collected by flushing the femur and tibia with PBS. These cells were centrifuged at 500 × g for 5 min. Whole blood was centrifuged at 500 × g, 4°C for 5 min and plasma was collected. The remaining blood cells and the resulting pellets were re-suspended in 1X red blood cell lysis buffer (Biolegend, San Diego, CA), at room temperature for 3 minutes followed by addition of 1 X PBS and centrifugation. Then, blood cells, spleen cells and bone marrow derived cells were stained with anti-CD11b, anti-7/4 and anti-Gr-1, stromal vascular fraction was stained with anti-CD11c and F4/80, both followed by incubation at room temperature for 45 minutes. Cells were subsequently washed with 1 X PBS and re-suspended in 1% neutral buffered formalin and run by flow cytometry (BD FACS LSR II™ flow cytometer, Becton Dickinson, San Jose, CA). Data was analyzed using BD FACS Diva software (Becton Dickinson, San Jose, CA). All antibodies were purchased from Biolegend, Miltenyi Biotec, or BD Bioscience.

### Quantitative RT-PCR

RT-PCR was performed using RNA extracted from different tissues of the experimental mice. Splenic cells from 8-week exposed KKay mice were seeded in one 24-well plate with 1.0 X 10^6^/well and treated with anti-CD3 (BD Biosciences, San Jose, CA) at a concentration of 1 μg/ml for different period. Cells were collected at the end of incubation. Total RNA from brown adipose tissue or splenic cells was isolated with Trizol (Invitrogen, Carlsbad, CA, USA) according to the manufacturer’s protocol. cDNA was reversely transcribed using High Capacity cDNA Transcription kit (Applied Biosystems, Carlsbad, California, USA). Quantitative polymerase chain reaction (qPCR) was performed in duplicate using the lightcycler 480. “No template”, cDNA negative controls were included for each gene set in all PCR reactions to detect contamination. The expression level for each gene was calculated using the ΔCt method relative to β-actin. The sequences of all primers are listed in Table 1.

**Table 1:** Primers used for real-time PCR

**Primer**	**Forward oligonucleotides**	**Reverse oligonucleotides**
*UCP1*	ACTGCCACACCTCCAGTCATT	CTTTGCCTCACTCAGGATTGG
*PGC1*α	GAGAATGAGGCAAACTTGCTAGCG	TGCATGGTTCTGAGTGCTAAGACC
*PRDM16*	CAGCACGGTGAAGCCATTC	GCGTGCATCCGCTTGTG
*Cidea*	ATCACAACTGGCCTGGTTACG	TACTACCCGGTGTCCATTTCT
*Elovl3*	GATGGTTCTGGGCACCATCTT	CGTTGTTGTGTGGCATCCTT
*CPT1M*	TGCCTTTACATCGTCTCCAA	GGCTCCAGGGTTCAGAAAGT
*F4/80*	TGTCTGACAATTGGGATCTGCCCT	TTGCATGTTCAGGGCAAACGTCTC
*INFɣ*	GCTCTGAGACAATGAACGCT	AAAGAGATAATCTGGCTCTGC
*IL-4*	TCGGCATTTTGAACGAGGTC	GAAAAGCCCGAAAGAGTCTC
*TNF*α	TTCCGAATTCACTGGAGCCTCGAA	TGCACCTCAGGGAAGAATCTGGAA
*IL-6*	ATCCAGTTGCCTTCTTGGGACTGA	TAAGCCTCCGACTTGTGAAGTGGT
β*-actin*	TGTGATGGTGGGAATGGGTCAGAA	TGTGGTGCCAGATCTTCTCCATGT

### Wire myograph and pressurized myograph studies

After the mice were sacrificed by isoflurane inhalation, vascular function of thoracic aorta was evaluated as previously [9]. Briefly, aorta with adhesive tissue were dissected out and placed in a dissecting dish filled with icecold oxygenated Krebs solution of the following composition (in mM): NaCl 119, KCl 4.7, NaHCO_3_ 25, CaCl_2__2.5_, MgCl_2_ 1, KH_2_PO_4_1.2, and D-glucose 11. After careful removal of adherent connective tissue, the artery was cut into 2 mm-length ring segments. The ring was suspended between two stainless bins in a 5-ml chamber on a Multi Myograph (Danish, Myo Technology A/S, Denmark) as previously described. Krebs solution in the bathing chamber was constantly bubbled with 95% O_2_ - 5% CO_2_ and maintained at 37°C (pH 7.4). Following 60 mins equilibration, each ring was stretched to 5 mN, a determined optimal resting tone for the development of isometric contraction. After artery contractility was tested, phenylephrine (1 μM, submaximal concentration) was used to contract the rings, followed by dose response of acetylcholine (30 nM-10 μM) in aorta. The rings were then rinsed in pre-warmed, oxygenated Krebs solution several times until a stable resting tone returned and finally equilibrated for 60 mins. The resting tone was readjusted to 5mN if necessary. And then, insulin (0.1U/ml – 10 U/ml) -induced relaxation was tested.

For pressure myograph studies, function of segments of resistance mesenteric arteries (2nd order) was evaluated as described [10]. Briefly, artery rings were dissected in Krebs solution, and cannulated between two glass cannulas with tip diameter <100 μm in a chamber filled with Krebs solution bubbled by 95% O_2_/5% CO_2_ and maintained at 37°C (pH 7.4). The intraluminal pressure was monitored, and vessel diameter was recorded by a light-inverted microscope with video camera monitored with the automated software Myo-View software (Danish Myo, Denmark). For testing endothelial responsiveness, phenylephrine (5 μM) was added to induce stable contraction at 100 mmHg intraluminal pressure and various doses of acetylcholine was used to test endothelial response at constant shear. The same setting was used for all the groups. After washing the vessel at the end of this intervention, graded doses of phenylephrine was tested to assess response. At the end of experiment, passive dilation was achieved by changing the bathing solution to Ca^2+^ free Krebs solution. Data was expressed as % of maximal KCl tone for phenylephrine and as % of phenylephrine tone (5 μM) for acetylcholine.

### Data analysis

Data are expressed as means ± standard error of the mean unless otherwise indicated. Graphpad Prism software (Version 5) was used for Student’s *t* test when 2 groups (PM and FA group) were compared. Concentration-relaxation curves were analyzed by two-way ANOVA followed by Bonferroni’s post-tests. *P* value of <0.05 was considered statistically significant.

## Results

### Exposure characteristics

The mean daily ambient PM_2.5_ concentration at the study site in Columbus, OH, was 8.28 ± 0.65 μg/m^3^. The mean concentration of PM_2.5_ was 1.51 ± 0.38 μg/m^3^ in the filtered air chamber, and 102.9 ± 19.16 μg/m^3^ in the exposure chamber respectively. This represented 12.4-fold concentration over ambient levels (Additional file 1: Figure S1). For the purposes of convenience concentrated ambient PM_2.5_ exposure is referred to PM/PM_2.5_ in this manuscript unless specified otherwise. Detailed elemental characterization of the exposure environment is provided in Table 2. Many trace elements including S, Ba, Zn, Ca, Mn and Mg, were elevated.

**Table 2:** Elemental constituents from OASIS in Columbus December 2011 to February 2012 by ICP-MS

**Elements**	**Ambient PM**_**2.5**_		**Filtered Air**		**Concentrated PM**_**2.5**_	
	**Mean**	**SD**	**Mean**	**SD**	**Mean**	**SD**
S	700.1	169.1	BID	50.3	6124.7	3789.4
Ca	69.8	24.5	66.6	46.7	504.1	335.5
Na	60.4	16.2	41.1	28.5	353.2	218.1
Fe	42.8	25.0	24.3	27.0	341.9	246.2
K	38.7	18.2	24.2	20.7	255.3	182.0
Zn	24.3	17.8	9.4	14.6	205.5	201.8
Mg	17.2	7.7	11.0	6.1	122.2	76.8
Al	15.1	11.8	20.3	26.3	123.5	138.2
P	14.3	10.5	14.5	6.3	96.4	105.7
Pb	3.5	1.6	BID	0.5	24.9	17.7
Cu	2.5	1.2	1.6	2.5	18.3	11.9
Ba	2.2	0.9	0.7	0.7	17.2	10.9
Mn	1.6	0.7	0.5	0.4	12.9	9.4
Cr	2.0	0.4	4.4	1.8	5.9	3.0
Se	0.6	0.2	0.0	0.0	5.6	3.6
Ti	0.6	0.3	0.1	0.1	4.9	3.2
Sb	0.6	0.2	0.0	0.0	4.3	2.6
Sr	0.3	0.2	0.2	0.1	2.9	1.8
As	0.4	0.1	0.0	0.0	3.4	2.1
Mo	0.4	0.1	0.3	0.3	2.7	1.7
Ni	0.2	0.3	BID	0.6	1.7	1.8
V	0.2	0.1	0.0	0.0	1.4	1.0
Cd	0.1	0.0	0.1	0.0	0.9	0.6
Rb	0.1	0.0	0.0	0.0	0.6	0.4
Ce	0.0	0.0	0.0	0.0	0.4	0.4
La	0.0	0.0	0.0	0.0	0.2	0.2
Co	0.0	0.0	0.0	0.0	0.2	0.2

Concentrations in ng/m^3^.

BID: below instrument detection limit.

### Effect of PM_2.5_ on energy homeostasis in KKay mice

**Figure 1:**
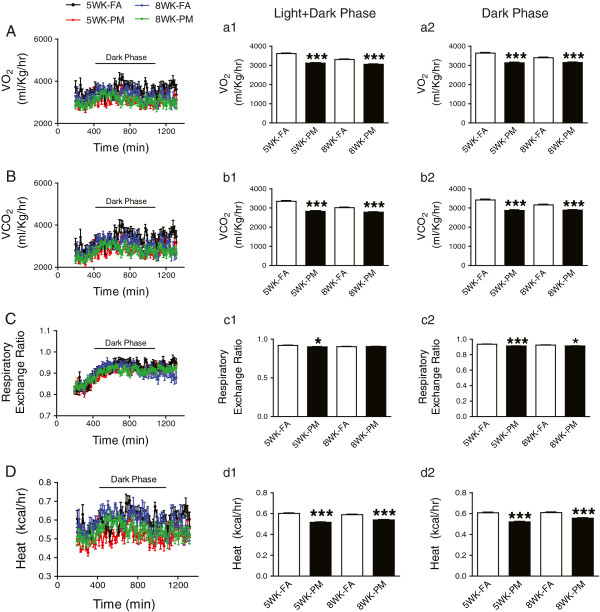
**Effect of PM**_**2.5 **_**exposure on energy homeostasis in KKay mice.** O_2_ consumption **(A)**, CO_2_ production **(B)**, respiratory exchange ratio **(C)** and heat production **(D)** of mice measured by indirect calorimetry over a 24 hrs. period (from 10:00 am to 10:00 am the next day). a1, b1, c1 and d1 were analysis for 24 hrs cycle (Light + Dark Phase); a2, b2, c2 and d2 were analysis for the dark phase. *P < 0.05, ***P < 0.001 compared to respective FA group. n = 6-8 per group.

Since KKay mice are well known to develop severe abnormalities in peripheral glycemic control and insulin over 10 weeks [11], we exposed 5-week old KKay mice to PM_2.5_ for 5- or 8-week periods and evaluated the whole body energy homeostasis. As shown in Figure 1, PM_2.5_ inhalation for 5 weeks significantly altered energy metabolism evidenced by decreased O_2_ consumption, CO_2_ production and heat production, *P* < 0.001 compared to respective FA group (Figure 1, A, B, D). Prolonged exposure (8 weeks) showed no further effect on these parameters (Figure 1, A-D). After 5 weeks exposure to PM_2.5_, we found significant decrease in respiratory exchange ratio, P < 0.05 compared to FA (Figure 1C). Although there was no difference between FA and PM groups in respiratory exchange ratio after 8 weeks PM_2.5_ exposure when the data from entire duration of 24 hrs (Light + Dark Phase) were analyzed, analysis data from the Dark phase showed significant decrement in respiratory exchange ratio (Figure 1C-c2).

### Effect of PM_2.5_ on glucose homeostasis in KKay mice

**Figure 2:**
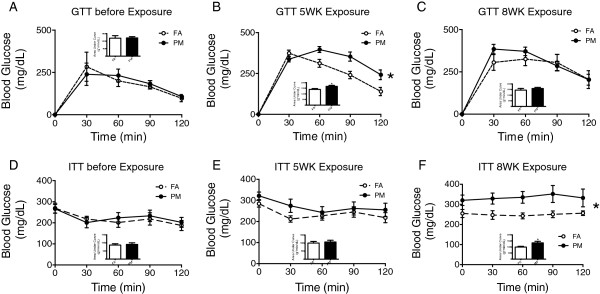
**Effect of PM**_**2.5 **_**exposure on glucose homeostasis in KKay mice. (A-C)**, The change of blood glucose in Glucose tolerance test (GTT) in overnight fasted mice before **(A)** and after 5-week **(B)** or 8-week **(C)** of PM_2.5_ exposure. **(D-F)**, Insulin tolerance test (ITT) in 4.5 hr fasted mice before **(D)** and after 5 wks **(E)** or 8 wks **(F)** of PM_2.5_ exposure. Bar graph is the AUC (area under curve) of the respective GTT or ITT curves. *P < 0.05, **P < 0.01 compared to respective FA group. n = 6-8 per group.

To test whether PM_2.5_ exposure detrimentally affects glucose control, we performed intra-peritoneal glucose tolerance test and insulin tolerance test in exposed mice. As shown in Figure 2A and 2D, there was no difference at baseline in glucose tolerance or insulin sensitivity prior to assignment to exposure. Mice showed significant elevations in glucose levels in response to intra-peritoneal glucose challenge, after 5-weeks of PM_2.5_ exposure but no significant difference after 8-weeks of exposure (Figure 2, B-C). Blood glucose was attenuated in response to insulin in PM_2.5_ exposed mice when compared to FA group after 5-weeks of exposure (Figure 2E). However, after 8-week exposure, standardized intra-peritoneal injection of insulin did not decrease blood glucose in either the FA or PM groups, with the blood glucose maintained at their initial levels during the 120-minute post-administration period (Figure 2F).

### Body and organ weight measurements

**Figure 3:**
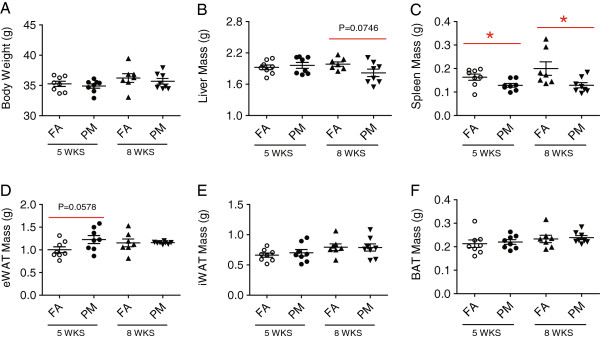
**Effect of PM**_**2.5 **_**exposure on body and organ weights in KKay mice.** Body weight **(A)**, liver weight **(B)**, spleen weight **(C)**, epidedymal white adipose tissue **(D)**, inguinal white adipose tissue **(E)** and brown adipose tissue **(F)** after 5 wks or 8 wks of PM_2.5_ exposure. *P < 0.05, **P < 0.01 compared to respective FA group. n = 7-8 per group.

Figure 3 illustrates the body and organ weights after 5-week or 8-week exposure to PM_2.5_ or FA. There were no significant differences in the body weight (Figure 3A) after the exposure. Interestingly, although the liver weight in PM_2.5_-exposed group was comparable to that in FA group after the 5-week exposure, mice in the 8-week PM_2.5_ exposure showed a clear trend to decrease when compared with the 5-week FA-exposed group (Figure 3B). The spleen weight was significantly lower in mice exposed to PM_2.5_ than that in FA-exposed mice at both 5-week and 8-week time points (Figure 3C). Although there was no difference in the weight of eWAT between 8WK-FA and 8WK-PM groups, a substantial trend (P = 0.0578) towards an increase in the adipose tissue weight was shown after 5-weeks of PM_2.5_ inhalation (Figure 3D). We also examined the weight of subcutaneous (inguinal) WAT (Iwat) and (interscapular) BAT and observed no effect of PM_2.5_ on fat weights in both the 8-week and 5-week exposed animals (Figure 3, E-F).

### Effect of PM_2.5_ exposure on circulating cytokines and adipokines

**Figure 4:**
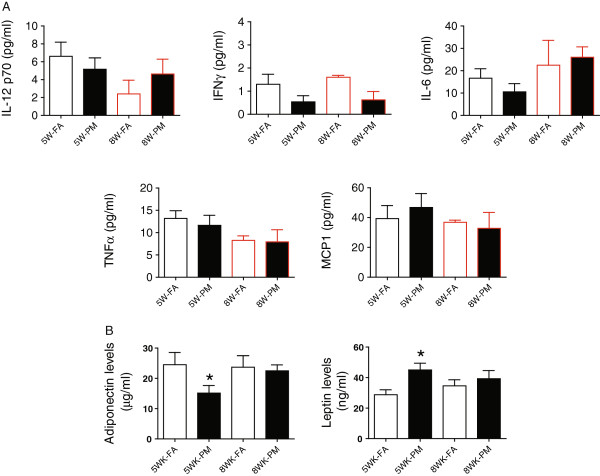
**The concentrations of circulating cytokines (IL-12p70, IFN-**γ**, IL-6, TNFα, and MCP-1) (A), adiponectin and leptin (B) in response to PM**_**2.5 **_**exposure.** *P < 0.05 compared to respective FA group. n = 7-8 per group.

We measured inflammatory biomarkers in the blood to see if PM_2.5_ exposure could exaggerate systemic inflammation. As shown in Figure 4A, we did not find any significant differences in plasma IL-12p70, IFN-γ, IL-6, TNFα, or MCP-1 between FA and PM groups at either the 5-week or 8-week periods. The levels of IL-10 were too low to be detectable. As shown in Figure 4B, the levels of plasma adiponectin were decreased by 5-week PM_2.5_ exposure accompanied by an increase in leptin levels when compared to FA controls (*P* < 0.05) suggesting alterations in this key adipokine in response to PM_2.5_ exposure [12]. There were no additional changes in adiponectin or leptin levels at the 8-week exposure (Figure 4B).

### Peripheral inflammation by PM_2.5_ in KKay mice

**Figure 5:**
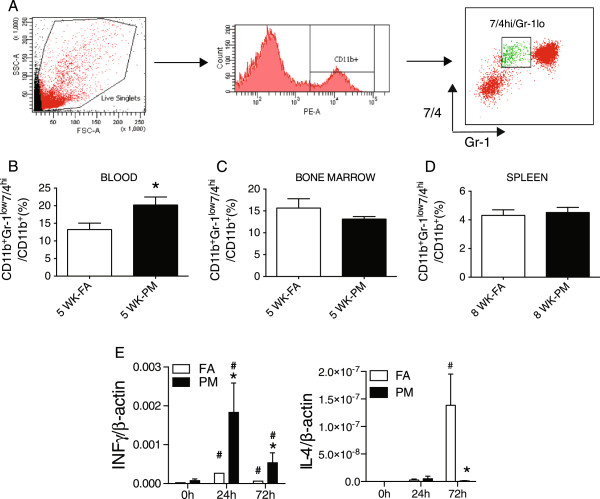
**Effects of PM**_**2.5 **_**exposure on inflammation in blood, bone marrow and spleen in KKay mice. (A)**, Identification of CD11b^+^Gr-1^low^7/4^hi^ cells from mice blood, bone marrow and spleen at the end of 5-week PM_2.5_ exposure. **(B-D)**, Analysis of the CD11b^+^Gr-1^low^7/4^hi^ population in blood **(B)**, bone marrow **(C)** and spleen **(D)**. **(E)**, mRNA levels of INFγ and IL-4 in cultured spleen cells treated with anti-CD3 at the end of 8 wks of PM_2.5_ exposure. *P < 0.05 compared to respective FA group, # *P* < 0.05 compared to respective group before treatment. n = 7-8 per group.

Because prior studies have demonstrated an increase in circulating inflammatory monocytes in response to air pollution exposure [13], we investigated this population of cells in PM_2.5_-exposed KKay mice. As shown in Figure 5, PM_2.5_ exposure increased CD11b^+^Gr-1^low^7/4^hi^ cells, the inflammatory monocytes, in the peripheral circulation (Figure 5B) with a corresponding trend towards a reduction in this population in the bone marrow (Figure 5C). Although there were no differences in splenic CD11b^+^Gr-1^low^7/4^hi^ cells (Figure 5D), IFNγ production from splenocytes of PM_2.5_-exposed mice was significantly higher compared to that of FA mice with a corresponding decrease in IL-4 release (Figure 5E). These results suggest a redirection of Th1/Th2 balance towards a Th1 polarized state in response to PM_2.5_ exposure.

**Figure 6:**
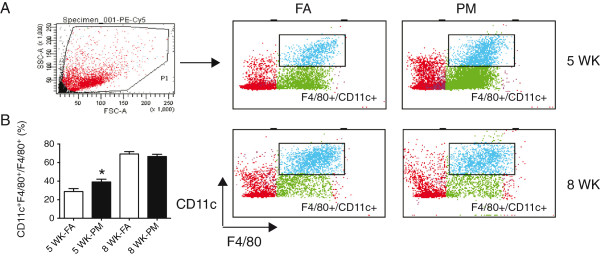
**Effects of PM**_**2.5 **_**exposure on inflammation in VAT in KKay mice. (A)**, Representative flow cytometric dot plots and analysis showing CD11c^+^F4/80^+^ cells from VAT at the end of PM_2.5_ exposure. **(B)**, Data were analyzed by relative percentage. *P < 0.05 compared to respective FA group. n = 7-8 per group.

We investigated inflammatory cell content in eWAT, which is regarded to play an important role in the development of IR, obesity, and type 2 diabetes mellitus [14-16]. In eWAT, we observed an increase in F4/80^+^/CD11c^+^ cells in response to 5-week PM_2.5_ exposure (Figure 6). Although there was no difference between the two groups after 8-weeks, this population in both groups surged to as high as 70% at 8 weeks, while this subset constituted 25-40% of the F4/80^+^/CD11c^+^ cells after 5-week PM exposure (Figure 6B).

### Brown adipocyte-specific and inflammation gene profiles expression in BAT

**Figure 7:**
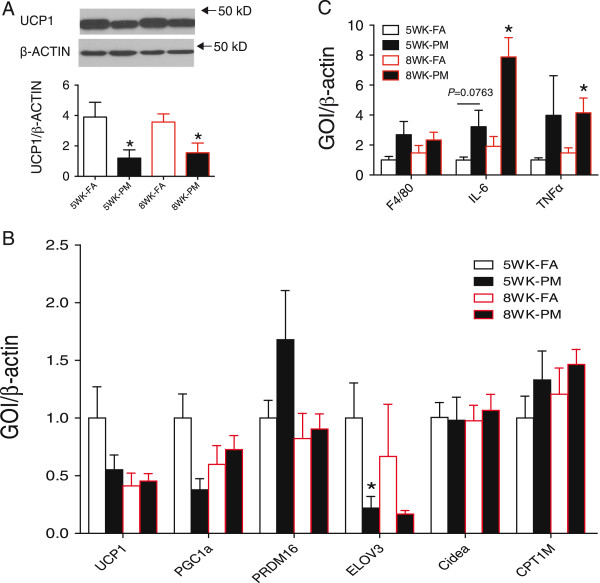
**Effect of PM**_**2.5 **_**exposure on BAT-specific gene profiles and UCP1 expression in KKay mice. (A)**, Altered protein levels of UCP1 in interscapular BAT in response to PM_2.5_ exposure. **(B)**, Altered mRNA levels of BAT-specific gene profiles in BAT in response to PM_2.5_ exposure. **(C)**, Expression of inflammation-related genes in BAT in response to PM_2.5_ exposure. *P < 0.05 compared to respective FA group. n = 6-8 per group. uncoupling protein 1 (*UCP1*), peroxisome proliferator-activated receptor-γ coactivator 1-α (*PGC-1*α), PRD1-BF1-RIZ1 homologous domain containing 16 (*PRDM 16)*, elongation of very long chain fatty acid 3 (*ELOV3*), Cell death-inducing DNA fragmentation factor--like effector A (*Cidea*), Carnitine palmitoyltransferase 1 muscle isoform *(CPT-1 M)*.

In light of the significant change in thermogenesis (Figure 1), we investigated uncoupling protein 1 (UCP1), a key player in thermogenesis via uncoupling of oxidative phosphorylation [17,18]. UCP1 protein (Figure 7A) but not mRNA level (Figure 7B) was down-regulated in interscapular BAT of PM_2.5_ exposed mice for both 5-week and 8-week exposure, consistent with decreased thermogenic activity (Figure 1). To determine brown adipocyte-specific gene profiles in diabetic mice in response to PM_2.5_ exposure, we examined several key genes related to mitochondrial function and regulatory networks that govern expression of genes during BAT differentiation and mitochondrial biogenesis by real-time PCR in the BAT. These genes include the expression of *Pgc-1*α (PPAR gamma coactivator 1α), *Prdm16* (a master regulator of brown adipocyte specification and differentiation), *CPT-1 M* (Carnitine palmitoyltransferase 1 muscle isoform), *Cidea* (a lipid droplet-associated protein with a role in fat storage) and *Elovl3* (an elongase enzyme important for elongation of monounsaturated or saturated very long chain fatty acids). However, no differences were observed except for the gene expression of Elovl3 was inhibited in response to PM_2.5_ exposure (Figure 7B). Although no significant upregulation of F4/80, was observed, an increase in IL-6 and TNFα was noted in BAT after 5-week or 8-week PM_2.5_ exposure (Figure 7C), indicating PM_2.5_ induced inflammation in BAT. Thus, alterations of UCP1 via post-transcriptional mechanisms and inflammation may account for the decreased thermogenesis and oxygen consumption in response to PM_2.5_ exposure.

### Defective insulin signaling in liver in response to PM_2.5_

**Figure 8:**
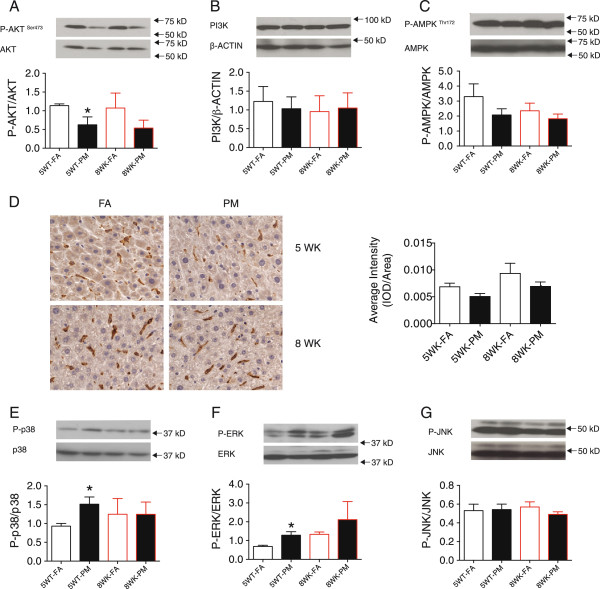
**Effects of PM**_**2.5 **_**exposure on inflammation, insulin and MAPK signaling pathways in the liver of KKay mice. (A-C)**, Western blotting for phosphorylated AKT (P-AKT)/total AKT **(A)**, PI3K **(B)**, and phosphorylated AMPK (P-AMPK)/total AMPK **(C)**. n = 3-7 per group. **(D)**, Representative image and analysis of F4/80^+^ staining by immunohistochemistry. n = 14-21 sections from 7–8 mice. **(E-G)**, Western blotting of signaling molecules involved in MAPK pathway [P-p38/p38, **(E)**; P-ERK/ERK, **(F)**; P-JNK/JNK, **(G)**]. n = 3-7 per group. *P < 0.05 compared to respective FA group.

As shown in Figure 8A, phosphorylated AKT (Ser473) was reduced in liver from PM_2.5_ group vs. respective FA group after 5-week exposure, without further alteration after 8-weeks of exposure. No alteration was seen in PI3K expression between all groups (Figure 8B). There was a slight trend towards decrease in the phosphorylation levels of AMPK (Thr172) in KKay mice with PM_2.5_ exposure at 5 and 8-weeks compared with FA controls (Figure 8C). Notably, the 8-week FA group showed decreased AMPK phosphorylation compared to the 5-week FA counterparts, indicating progressive worsening of disease in the mice over time. To further explore mechanisms by which PM_2.5_ impaired glucose homeostasis and hyperlipidemia, we assessed inflammatory signals implicated in hepatic IR. No further increase in F4/80 content in the liver of mice (Figure 8D) were noted with PM_2.5_ exposure, in excess of that noted in FA at both time points. Immunoblotting analysis demonstrated increased levels of activated p38 and ERK, but not JNK, in the liver of the mice exposed to PM_2.5_ for 5-weeks, compared to that in the mice exposed to FA (Figure 8, E-G). Compared to 5WK-FA group, there was a trend towards an increase in levels of p38 and ERK in the liver of both FA mice and PM mice exposed for 8 weeks. However, we observed no significant change in the 8WK-PM group compared to that in FA group (Figure 8, E-F).

### PM_2.5_ does not exacerbate endothelial dysfunction in KKay mice

Exaggerated constriction of small mesenteric arterioles in response to phenylephrine with no alteration in response to acetylcholine was noted with PM_2.5_ exposure (Additional file 1: Figure S2A). Responses to acetylcholine and insulin mediated vasodilation in conduit arterial segments (aorta) were also profoundly diminished, consistent with marked hyperglycemia and hyperinsulinemia in the KKay model with no further alteration after PM_2.5_ exposure (Additional file 1: Figure S2B).

## Discussion

In this paper we evaluated the role of ambient air pollution on energy metabolism, glucose homeostasis, and inflammatory signaling pathways in a genetically predisposed model that develops severe Type II DM within 6–10 weeks. There are several important findings in this study that support an adjunctive role of air-pollution exposure in potentiating development of Type II DM. Firstly, PM_2.5_ exposure had effects on energy metabolism including reduction of O_2_ consumption, CO_2_ production, respiratory exchanging ratio and heat generation. Secondly, PM_2.5_ exposure exaggerated IR, visceral adiposity and peripheral inflammatory response. Thirdly, PM_2.5_ exposure resulted in lowered UCP1 expression in BAT together with increased expression of inflammatory genes. Finally, PM_2.5_ exposure resulted in activation of p38 and ERK but not JNK in the liver of KKay mice.

There is increasing evidence suggesting links between exposure to environmental toxins and susceptibility to Type II DM. Consistent with the study in a diet-induced obesity model [6,8,19], PM_2.5_ elevated blood glucose levels and impaired insulin signaling evidenced by reduced Akt phosphorylation in the genetic KKay mice. These results indicate the reproducible effect of air pollution in mediating adverse metabolic consequences in a genetically susceptible model of Type II DM. Thus it provides additional experimental evidence to support the association between PM_2.5_ and cardiometabolic disease.

One of the central findings of this study is the changes in metabolic indices demonstrated in carefully performed metabolic cage studies. Recent studies have suggested a compelling role for BAT in regulating metabolism and insulin resistance [20-22]. Brown adipocytes play an important role in dissipation of energy in the form of heat, a process called non-shivering thermogenesis. A reduction in thermogenic function of BAT has been linked to the development of IR and obesity [20-22]. UCP1, which is specifically expressed in BAT mitochondria, is largely responsible for the uncoupling of respiration from ATP synthesis resulting in dissipation of energy as heat, playing a pivotal role in thermogenesis [23]. We have previously demonstrated abnormalities in BAT structure and function with chronic exposure to PM_2.5_[7]. These changes include electron microscopic changes in BAT mitochondria as well as transcriptional reprogramming of BAT genes which include UCP1 expression.

The substantial down-regulation of UCP1 protein in BAT in the PM_2.5_ exposed mice may potentially account for decreased heat production and thereby explain reduction in O_2_ consumption and CO_2_ production. Interestingly, high fat diet treated mice showed substantial compensatory increase in the expression of UCP1 both at the protein levels and mRNA levels, likely representing an adaptive mechanism to excess caloric intake [24]. Fromme et al. summarized 62 relevant studies of which 42 studies have previously demonstrated that high fat diet itself enhanced UCP1 expression [25]. Thus our findings suggest a unique role of PM_2.5_ exposure in preventing the adaptive increase of UCP1. These findings are consistent with our previous studies showing that long term PM_2.5_ exposure reduced UCP1 expression in C57B/L6 mice [7]. An important point in our study that deserves further explanation is the finding that in many end-points we observed a difference at 5 weeks but not at 8 weeks. It is possible that the severity of phenotype at 8 weeks in the KKay makes it very difficult to distinguish end-points at 8 weeks between groups.

White adipose tissue is a major source of energy for the human body. The increased epididymal (visceral) fat mass in mice exposed to concentrated PM_2.5_ demonstrated that PM_2.5_ inhalation induced visceral adiposity likely through adipocyte hypertrophy (indicating excess energy storage which was supported by decreased energy expenditure in PM_2.5_-exposed KKay mice) and enhanced inflammatory cell infiltration. Macrophages in the adipose tissue have been shown to increase from 10-15% to 45-60% of total cellular content during obesity and may independently have contributed to expansion of VAT mass [26]. In addition, the adipose tissue is also a source of major adipocytokines such as adiponectin and leptin. The expression of adiponectin decreased with increase in the adiposity and reduction of adiponectin has been associated with insulin resistance, dyslipidemia, and atherosclerosis in humans [27]. In line with this, circulating adiponectin levels were significantly reduced by PM_2.5_ inhalation as has been shown by us previously [6]. Adiponectin plays an important role in mediating insulin-sensitization through binding to its receptors AdipoR1 and AdipoR2, leading to activation of adenosine monophosphate dependent kinas (AMPK) and presumably other yet-unknown signaling pathways. A trend towards a decrease in AMPK phosphorylation in liver of the KKay mice exposed to PM_2.5_ could potentially be attributable to reduced adiponectin release. Leptin is another major adipokine from adipose tissue. Contrary to the remarkably decreased circulating leptin levels in response to PM_2.5_ exposure in C57BL/6 mice fed on regular chow [7], we observed an increase in leptin levels in the PM_2.5_ inhaled KKay mice. The mechanisms by which leptin expression is regulated need further study. The net action of leptin is to inhibit appetite, stimulate thermogenesis, enhance fatty acid oxidation, decrease glucose, and thus reduce body weight and fat through central mechanisms [27]. Recent studies have suggested that central leptin resistance may contribute to attenuation of the well known effects of leptin. Since the circulating leptin levels in our studies were increased in response to PM_2.5_ exposure, our results raise the possibility of leptin resistance as an additional component and will need further study. Consistent with our findings, Bremer et al. demonstrated the novel observation that adipose tissue in subjects with nascent metabolic syndrome have increased levels of leptin as well as decreased levels of adiponectin and omentin-1, concomitant with increased adipokines such as IL-1, IL-6, IL-8, PAI-1 and MCP1 [28,29]. Thus it can be assumed that adipose tissue dysregulation and aberrant adipokine secretion contribute towards the syndrome’s low-grade chronic proinflammatory state and IR accompanied by correction for increased adiposity in the current KKay mice.

A number of studies have highlighted the innate immune mechanisms as the critical role that is responsible for the pathophysiological abnormalities, including IR. In line with this, our results demonstrated that although PM_2.5_ inhalation did not enhance inflammotary cytokines production in blood, it did result in increased circulating inflammatory monocytes (CD11b+/7/4hi/Gr-1low) accompanied by a corresponding reduction in bone marrow. We have previously demonstrated this same finding in our earlier studies and shown an important role for TLR4 in mobilization of inflammatory subsets of monocytes in response to PM_2.5_ exposure [13]. This subset is believed to mediate pro-inflammatory effects and a decrease in this population has been associated with favorable end-points including regression of atherosclerotic lesions and macrophage accumulation [30]. It is well known that monocytes originate from progenitors in the bone marrow and traffic via the bloodstream to peripheral tissues [31]. As a homeostatic response to diverse triggers, circulating monocytes leave the bloodstream and migrate into tissues where, they differentiate into macrophage or dendritic cell populations following conditioning by local growth factors such as pro-inflammatory cytokines and microbial products [31]. F4/80^+^/CD11c^+^ is a widely used marker to label “classically activated” macrophages that have been demonstrated to play a pathophysiological role in high-fat diet-induced obesity [26,32-34]. Our findings in this study extend our previous observations where we have shown increased macrophage infiltration into VAT in response to concentrated PM_2.5_ exposure, in excess of the effects of high-fat diet alone [6]. Furthermore, the macrophages in VAT in mice exposed to PM_2.5_ (72.7 μg/m^3^, 128 days) demonstrated an “M1” profile with increased cytokines such as TNFα and IL-6, and reduced expression of IL-10 and *N*-acetyl-galactosamine specific lectin 1 [6]. Using a mouse model with yellow fluorescent protein (YFP)-expressing monocytes (c-*fms*^YFP^), we further confirmed enhanced monocyte adhesion in microcirculation of VAT and accumulation in visceral fat [6]. The increased monocyte/macrophage infiltration in VAT in response to PM_2.5_ exposure further appears to be dependent on CCR2 (116.9 ± 34.2 μg/m^3^, ~17 weeks), as it was abolished by genetic ablation of CCR2 [19]. Thus, the increased population of F4/80^+^/CD11c^+^ in eWAT suggest mechanisms similar to those involved in diet mediated aggravation of the VAT infiltration via CCR2-dependent pathways [33]. Except for the increase in excess oxidative and nitrosative stress in BAT after long term PM_2.5_ exposure [7], we demonstrated in the current study that PM_2.5_-mediated up-regulation of pro-inflammatory genes in BAT and provided additional explanation for the inhibited energy metabolism. Together with the redirection of Th1/Th2 balance towards a Th1 polarized state in response to PM_2.5_ exposure (Figure 5B), these results suggest the increased inflammation in visceral WAT, BAT and Th1 polarization in spleen indicated recruitment of monocytes into tissues, therefore, contributed to the pathogenesis of inflammatory diseases such as IR or diabetes.

p38 MAPK belongs to a family of evolutionarily conserved serine-threonine MAPKs that link extracellular signals to intracellular machinery regulating a plethora of cellular processes. Together with JNK, they are activated by environmental or genotoxic stress and described as stress-activated protein kinases [35-37]. Different from the observation with PM_2.5_-exposed C57BL/6 mice fed on regular chaw by Zheng et al. [38], we found activation of p38 and ERK but not JNK in response to PM_2.5_ in the KKay mice. These differences likely represent differences in strains and genetic susceptibility, in addition to the complex interactions of diet and environmental signals. In keeping a line with our study, Jiao et al. have suggested that the increased hepatic ERK activity may contribute to increased liver glycogen content and decreased energy expenditure in obesity and may play a central role in hepatic glucose and lipid metabolism [39,40]. However, whether increased MAPKs activity is causal or a homeostatic consequence remains to be determined as others have suggested that increases in p38 activity may regulate Xbp1 nuclear translocation and activity and thus may represent a compensatory mechanism to maintain homeostatic response [41]. Therefore, the significance of these findings and precise role of p38 warrants further studies.

In summary, our results suggest that particulate air pollution exposure resulted in dysregulated metabolism and influenced IR likely through complex pathways involving the liver, visceral and brown adipose tissue in a genetic KKay diabetes model. These findings suggest an important role for PM_2.5_ in modulating susceptibility to Type II DM and may have important implications for public health at a global scale.

## Abbreviations

BAT: Brown adipose tissue; FA: Filtered air; IR: Insulin resistance; ITT: Insulin tolerance test; IPGTT: Intra-peritoneal glucose tolerance testing; PM: Particulate matter; PM_2.5_: Particulate matter <2.5 μm in aerodynamic diameter; Type II DM: Type II diabetes; UCP1: Uncoupling protein 1; eWAT: Epididymal white adipose tissue.

## Competing interests

The authors declare that they have no competing interests.

## Authors’ contributions

CL, YB, XX, AW, TW, ZY, SM and MM performed the experiments and contributed to acquisition of data. CL, ZY, and SM analyzed the data and interpreted the results. AW contributed to PM_2.5_ exposure of the animals. The manuscript was written by CL and revised critically by SR, QS, LS, MP and JTD. All authors read, corrected and approved the manuscript.

## Supplementary Material

Additional file 1: Figure S1 PM_2.5_ concentration to which mice were exposed at the study site. **Figure S2.** Effect of PM_2.5_ exposure on vascular function from KKay mice. A, Dose–response to phenylephrine, acetylcholine and intraluminal pressure in small mesenteric artery at the end of PM_2.5_ exposure. B, Dose response to acetylcholine and insulin in in aortic rings precontracted with phenylephrine at the end of PM_2.5_ exposure. *P < 0.05 compared to FA group. n = 5-8 per group. Click here for file
