# Supplementary figures and images for: Exaggerated effects of particulate matter air pollution in genetic type II diabetes mellitus
Source: Part Fibre Toxicol. 2014 May 30;11:27. doi: 10.1186/1743-8977-11-27 (PMC4049808; doi:10.1186/1743-8977-11-27)

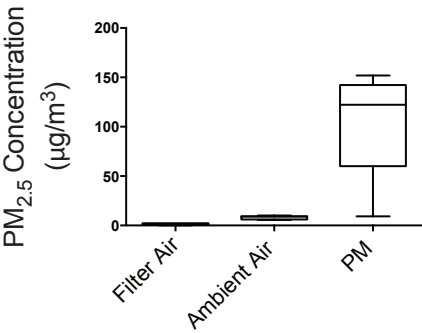

A

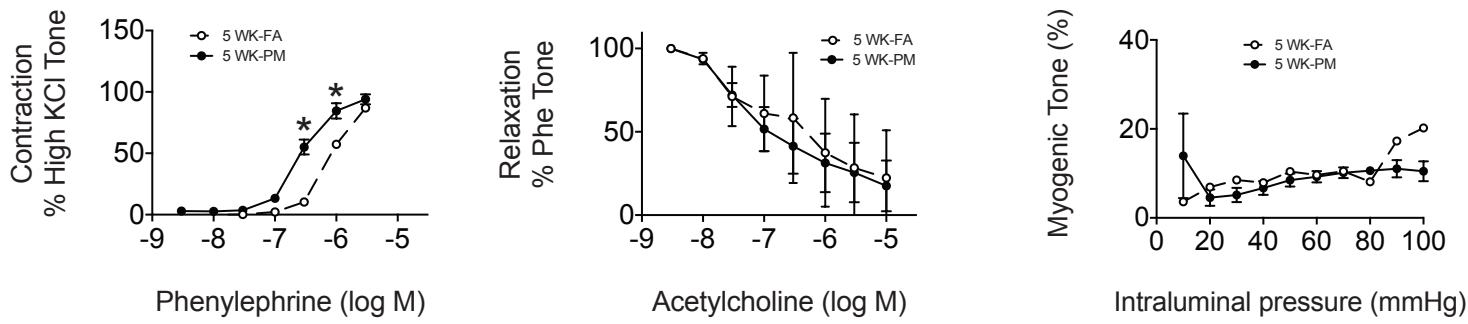

B

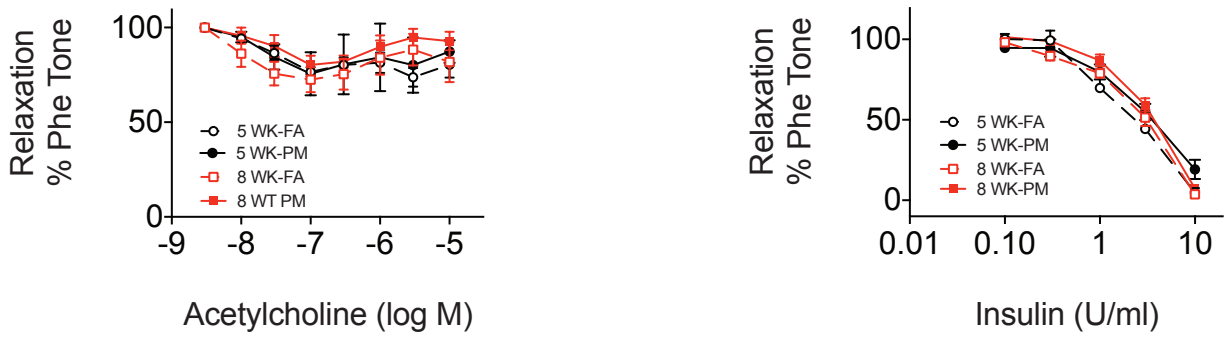

Supplement: Additional file 1: Figure S1 — PM2.5 concentration to which mice were exposed at the study site. Figure S2. Effect of PM2.5 exposure on vascular function from KKay mice. A, Dose–response to phenylephrine, acetylcholine and intraluminal pressure in small mesenteric artery at the end of PM2.5 exposure. B, Dose response to acetylcholine and insulin in in aortic rings precontracted with phenylephrine at the end of PM2.5 exposure. *P < 0.05 compared to FA group. n = 5-8 per group. [file 1743-8977-11-27-S1.pdf]
